# Analysis of PPI networks of transcriptomic expression identifies hub genes associated with Newcastle disease virus persistent infection in bladder cancer

**DOI:** 10.1038/s41598-022-20521-z

**Published:** 2023-05-05

**Authors:** Umar Ahmad, Syahril Abdullah, De Ming Chau, Suet Lin Chia, Khatijah Yusoff, Soon Choy Chan, Teng Aik Ong, Azad Hassan Razack, Abhi Veerakumarasivam

**Affiliations:** 1 Medical Genetics Laboratory, Genetics and Regenerative Medicine Research Centre, Faculty of Medicine and Health Sciences, Universiti Putra Malaysia, 43400 UPM Serdang, Selangor Malaysia; 2 Medical Genetics Unit, Faculty of Basic Medical Sciences, Bauchi State University, Gadau, PMB 65, Itas/Gadau, Nigeria; 3 MAKNA Cancer Research Laboratory, Institute of Bioscience, Universiti Putra Malaysia, 43400 UPM Serdang, Selangor Malaysia; 4 Department of Microbiology, Faculty of Biotechnology and Biomolecular Sciences, Universiti Putra Malaysia, 43400 UPM Serdang, Selangor Darul Ehsan Malaysia; 5 Malaysia Genome Institute, Ministry of Science, Technology and Innovation, Jalan Bangi, 43000 Kajang, Selangor Darul Ehsan Malaysia; 6 School of Liberal Arts, Science and Technology (PUScLST), Perdana University, Perdana University, 50490 Kuala Lumpur, Malaysia; 7 Department of Surgery, Faculty of Medicine, University of Malaya, Wilayah Persekutuan, Kuala Lumpur, Malaysia; 8 Department of Biological Sciences, School of Medical and Life Sciences, Sunway University, 47500 Bandar Sunway, Selangor Darul Ehsan Malaysia

**Keywords:** Cancer, Computational biology and bioinformatics, Genetics, Biomarkers

## Abstract

Bladder cancer cells can acquire persistent infection of oncolytic Newcastle disease virus (NDV) but the molecular mechanism(s) remain unelucidated. This poses a major barrier to the effective clinical translation of oncolytic NDV virotherapy of cancers. To improve our understanding of the molecular mechanism(s) associated with the development of NDV persistent infection in bladder cancer, we used mRNA expression profiles of persistently infected bladder cancer cells to construct PPI networks. Based on paths and modules in the PPI network, the bridges were found mainly in the upregulated mRNA-pathways of p53 signalling, ECM-receptor interaction, and TGF-beta signalling and downregulated mRNA-pathways of antigen processing and presentation, protein processing in endoplasmic reticulum, completement and coagulation cascades in persistent TCCSUPPi cells. In persistent EJ28Pi cells, connections were identified mainly through upregulated mRNA-pathways of renal carcinoma, viral carcinogenesis, Ras signalling and cell cycle and the downregulated mRNA-pathways of Wnt signalling, HTLV-I infection and pathways in cancers. These connections were mainly dependent on *RPL8-HSPA1A*/*HSPA4* in TCCSUPPi cells and *EP300*, *PTPN11*, *RAC1*—*TP53*, *SP1*, *CCND1* and *XPO1* in EJ28Pi cells. Oncomine validation showed that the top hub genes identified in the networks that include *RPL8*, *THBS1*, *F2* from TCCSUPPi and *TP53* and *RAC1* from EJ28Pi are involved in the development and progression of bladder cancer. Protein-drug interaction networks identified several putative drug targets that could be used to disrupt the linkages between the modules and prevent bladder cancer cells from acquiring NDV persistent infection. This novel PPI network analysis of differentially expressed mRNAs of NDV persistently infected bladder cancer cell lines provide an insight into the molecular mechanisms of NDV persistency of infection in bladder cancers and the future screening of drugs that can be used together with NDV to enhance its oncolytic efficacy.

## Introduction

Bladder cancer (BC) arises from the bladder epithelial lining and can subsequently spread into and beyond the muscle layer of the bladder^1^. It is the tenth most common cancer in the world and accounts for 4.7% of all new cancer cases^2^. The poor prognosis of BC is due to the limited treatment options for advance disease and resistance to conventional therapies. Newcastle disease virus (NDV) is a promising putative cancer-specific agent that can kill human tumour cells while sparing normal cells ^3–5^. This selective killing of cancer cells by NDV is due to the defects in antiviral responses such as the production of interferons that favour viral replication^6^. The mechanism by which NDV kills cancer cells is via the activation of both intrinsic and extrinsic apoptosis pathways^7^ and direct virus-mediated oncolysis^8^. Moreover, NDV is able to trigger a long-term adaptive immune-response against infected cancer cells^9^, making it a strong inducer of cancer cell death. However, NDV has also been found to persistently infect subpopulations of cancer cells that are able to resist NDV-mediated oncolysis^10^. Similar observations have been reported in other oncolytic viruses such as Reovirus^11^ and Measles virus^12^. Persistent infection indicates the ability of a subpopulation of cancer cells to resist NDV-mediated oncolysis. Thus, persistent infection in cancer cells poses a potential barrier to clinical translation especially when tumours typically contain heterogenous subsets of cells that harbour a spectrum of genetic aberrations. Although recombinant NDV could possess enhanced oncolytic capabilities, the risk of persistent infection still remains. The exact mechanism by which cancer cells acquire NDV persistent infection and the molecular basis underlying the development of persistent infection in BC have not been completely elucidated.

To unravel the regulatory mechanisms of oncolytic NDV persistent infection in bladder cancer at a molecular level, we previously compared the mRNA expression profiles of normal and cancer cells of the human bladder using transcriptomics^13^. A total of 63 and 134 differentially regulated genes were identified in  TCCSUPPi and EJ28Pi (persistently infected cells) relative to their control cells respectively;  whereby 25 genes were upregulated (log2 fold-change ≥ 0) and 38 genes were downregulated (log2 fold-change ≤ 0) in TCCSUPPi cells. Whereas, 55 genes were upregulated (log_2_ fold-change ≥ 0) and 79 genes were downregulated (log_2_ fold-change ≤ 0) in EJ28Pi cells^13^. These differentially expressed genes (DEGs) were significantly enriched in some important upregulated pathways such as TGF-beta signaling, KRAS signaling up and interferon gamma response. Evasion of apoptosis was identified as crucial for the development of persistent infection in bladder cancer cells. The data further revealed that some essential molecular functions such as calcium binding (GO:0005509) and DNA-binding transcription repressor activity, RNA polymerase II-specific (GO:0001227) in TCCSUPPi and protein domain specific binding (GO:0019904) and RNA polymerase II regulatory region sequence-specific DNA binding (GO:0000977) were significantly enriched in EJ28Pi. Together, these gene expression changes may cohesively contribute towards the development of NDV persistent infection in bladder cancer.

Proteins function collectively via protein–protein interactions (PPI) within the cell. These interactions are essential for most biochemical activities to achieve their specific function(s) in a living cell^14,15^ and to provide single proteins with multiple functions^16,17^. Thus, employing PPI methodologies to unravel the orchestrated and/or stochastic molecular mechanisms of biological processes have drawn increasing attention in recent time^15,18,19^. To deeply understand the regulatory mechanisms underlying the state of many diseases, PPI networks are generally performed by analysing the DEGs obtained from those disease states^17,20,21^. However, there is currently no PPI network analysis for DEGs obtained from established persistently infected bladder cancer cells. In this study, the DEGs associated with TCCSUPPi (63) and EJ28Pi (134) were analysed separately to construct a PPI network that will enable a better understanding of the molecular mechanism(s) underlying the development of NDV persistent infection in bladder cancer.

## Results

### PPI network of DEGs from TCCSUPPi

**Figure 1:**
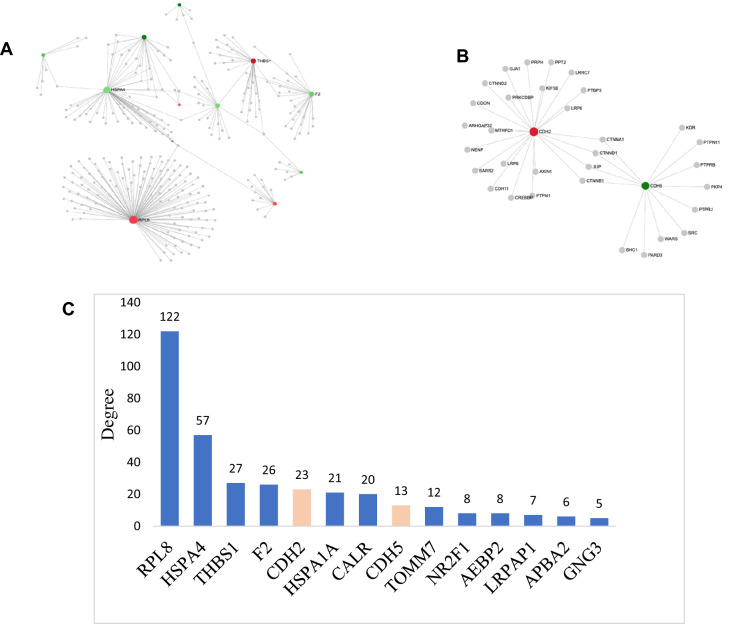
First and second networks of TCCSUPPi cells. (**A**) First identified network of TCCSUPPi cells. The red and green colours represent the nodes expression, that are up- and down-regulated, respectively. The expression levels are represented by the shades of colour and the area of the nodes indicate the degrees in which the nodes are connected to each other. Nodes with gene names are the top 4 nodes in the PPI network. (**B**) Second identified network in TCCSUPPi cells. Subnetwork 2 contains both up- and downregulated nodes that are affected in the pathways. Nodes in red and green colour are upregulated and downregulated in TCCSUPPi cells, respectively. (**C**) Hub nodes in the PPI network of TCCSUPPi cells. Top 14 hub nodes with their degree levels. Genes in blue colour are from subnetwork 1 and red from subnetwork 2. The PPI network figures A & B were generated using a multifunctional online software, Network Analyst (https://www.networkanalyst.ca)^23,24^.

To better understand the regulatory mechanisms employed by bladder cancer cells that enable the development of NDV persistent infection, differentially expressed genes (DEGs) associated with persistent TCCSUPPi and EJ28Pi cell lines were used to construct protein–protein interactions (PPI) network(s) through STRING Interactome database^22^. All the DEGs associated with TCCSUPPi (63) and EJ28Pi (134) were analyzed separately for network interactions. As a result, 6 subnetworks that included a continent (subnetwork 1) and 5 islands (subnetwork 2–6) were identified in TCCSUPPi cells. Two subnetworks with highest scores were selected for further analysis. The subnetwork 1 contained 291 nodes, 309 edges and 12 seeds (Fig. 1A) and subnetwork 2 had 34 nodes, 36 edges and 2 seeds (Fig. 1B). The expression levels and degrees of connection between nodes were represented by colours and areas respectively. The hub nodes in the entire network were further analyzed and the top 14 hub nodes were selected and graphically presented (Fig. 1C). Twelve (12) hub nodes out of 14 were identified to be mostly from subnetwork 1 and two superfamily member of cadherin (*CDH2* and *CDH5*) were clustered in subnetwork 2 (Fig. 1B) with *CDH2* upregulated and *CDH5* downregulated.

### Functional connections in the network

**Figure 2:**
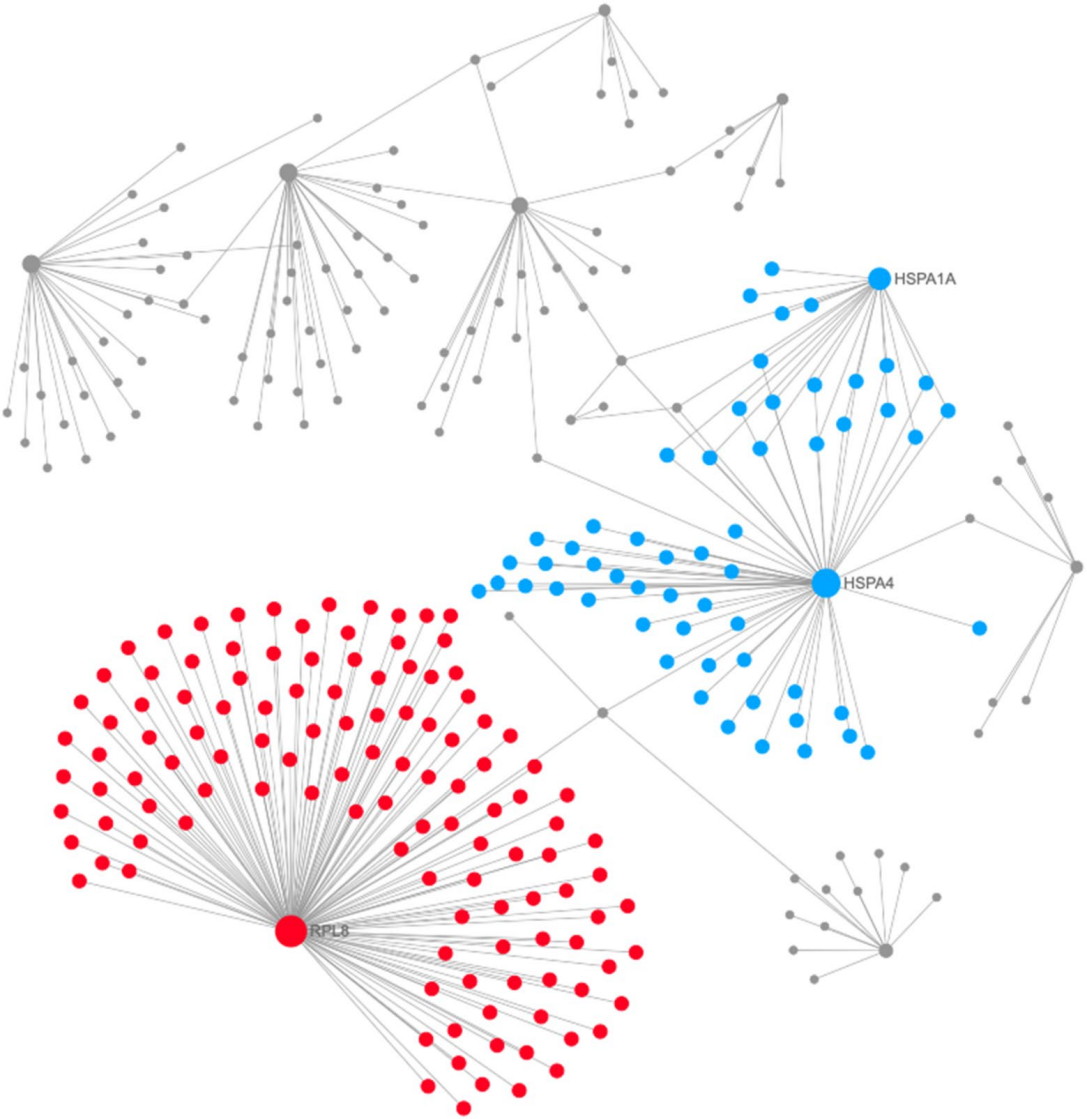
Modules 0 and 1 in the TCCSUPPi PPI network. The modules in blue and red are module 0 and module 1, respectively. The degrees of the nodes that connect to others in the network are represented by the areas of the nodes. The PPI network figure is generated using a multifunctional online software, Network Analyst (https://www.networkanalyst.ca)^23,24^.

Connections between functions in the identified networks were further explored and the related nodes were re-constructed (Fig. 2). As illustrated, pathways of bladder cancer, malaria, mitophagy, p53 signaling, ECM-receptor interaction, TGF-beta signaling, phagosome, ribosome, focal adhesion and proteoglycans in cancer were significantly enriched (*p* < 0.05) in the upregulated DEGs in the nodes connecting the PPI network (subnetwork 1) (Supplementary Table S1). Antigen processing and presentation, protein processing in endoplasmic reticulum, prion diseases, legionellosis, longevity regulating, complement and coagulation cascades, platelet activation and spliceosome pathways were significantly enriched (*p* < 0.05) in the downregulated DEGs with connected nodes in the PPI network (subnetwork 1) (Supplementary Table S2).

To identify the nodes that are implicated in the aforementioned pathways, medullary analysis was carried out. A total of 9 functional clustered modules and related hub genes were discovered.  However, only modules with majority of the nodes were used for redesigning of the modular network. Modules 0 and 1 were observed to contain a significant number of the nodes (*p* < 0.05) that contributed to the activation of pathways mentioned above. The top two significant modules were presented in different colours (Fig. 2). The results demonstrate that module 0 (coloured blue) and module 1 (coloured red) are key players in in the PPI network of the TCCSUPPi cells, which means that the cluster of genes in modules 0 and 1 act together to promote the development of NDV persistent infection in TCCSUP bladder cancer cell line. The results further illustrate how the upregulated *RPL8* and downregulated *HSPA1A*/*HSPA4* are functionally connected (Fig. 2).

### Protein drug interactions in TCCSUPPi

**Figure 3:**
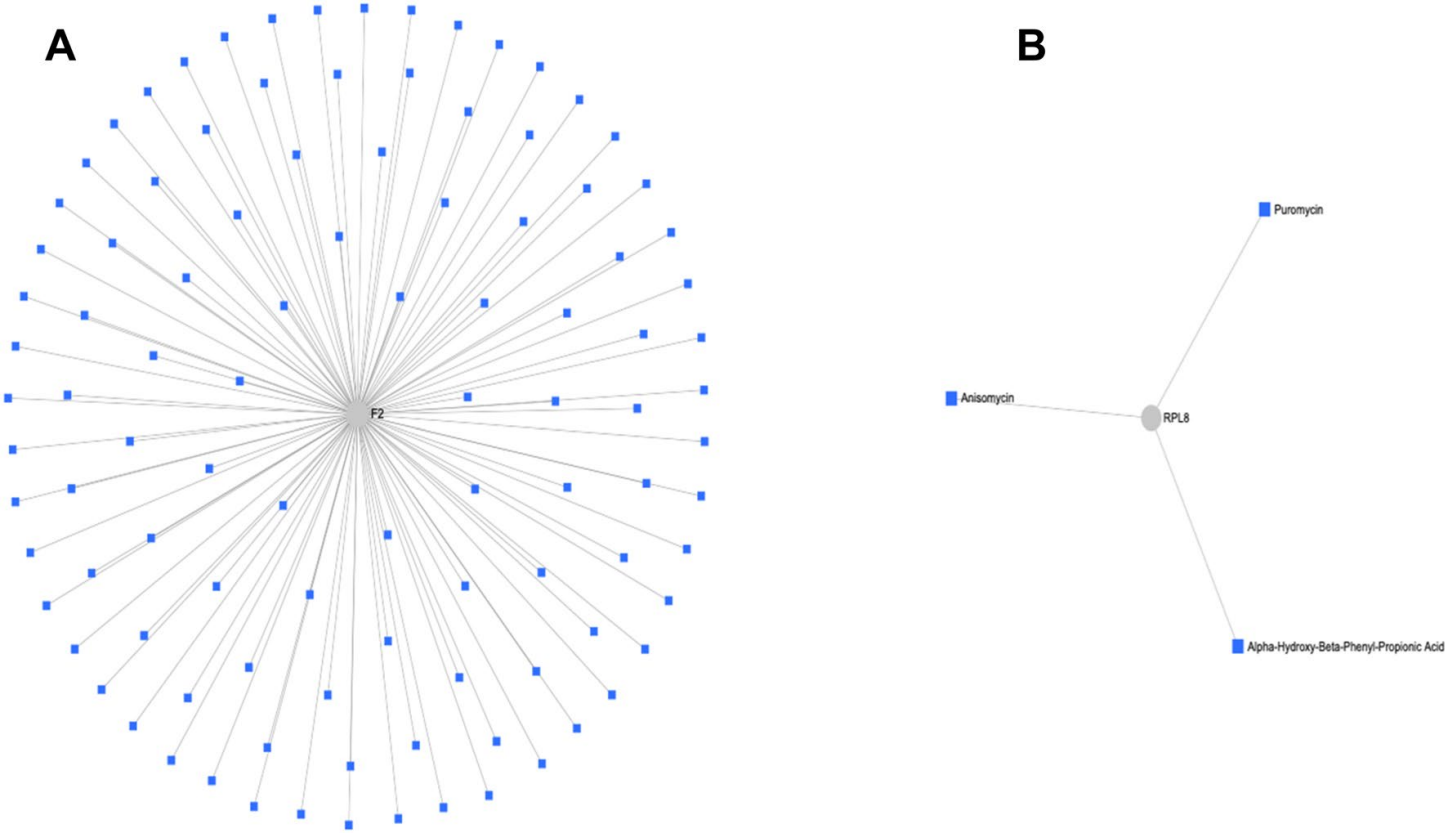
Protein-drug interaction network. (**A**) The figure illustrates interactions between the downregulated node (*F2*) and several multiple drugs. (**B**) The figure shows interactions between the upregulated node (*RPL8*) and three drugs. The PPI network figure (**A**,**B**) were generated using a multifunctional online software, Network Analyst (https://www.networkanalyst.ca)^23,24^.

To identify drug interactions between these connected nodes, we carried out protein-drug interaction analysis using the upregulated nodes that included *RPL8* and *THBS1* and downregulated nodes that included *F2* and *HSPA4*. Two subnetworks were identified. Subnetwork 1 comprises of 104 nodes, 103 edges and 1 seed while subnetwork 2 has 4 nodes, 3 edges and 1 seed. Based on the analysis of subnetwork 1, several drugs were identified to be connected to the coagulation factor II, thrombin (*F2*) node. The top major drugs that are linked to *F2* are lepirudin, bivalirudin, drotrecogin alfa, coagulation factor IX (recombinant), menadione, argatroban, and proflavine (Fig. 3A). The remaining list of drugs can be found in the appendix. In subnetwork 2, ribosomal protein L8 (*RPL8*) that was upregulated is linked to alpha-hydroxy-beta-phenyl-propionic acid, anisomycin and puromycin drugs (Fig. 3B). These results demonstrate that the identified drugs above can be used to potentially suppress the upregulated and downregulated nodes, making it possible to prevent TCCSUP bladder cancer cells from acquiring NDV persistent infection.

### PPI network of DEGs from EJ28Pi

**Figure 4:**
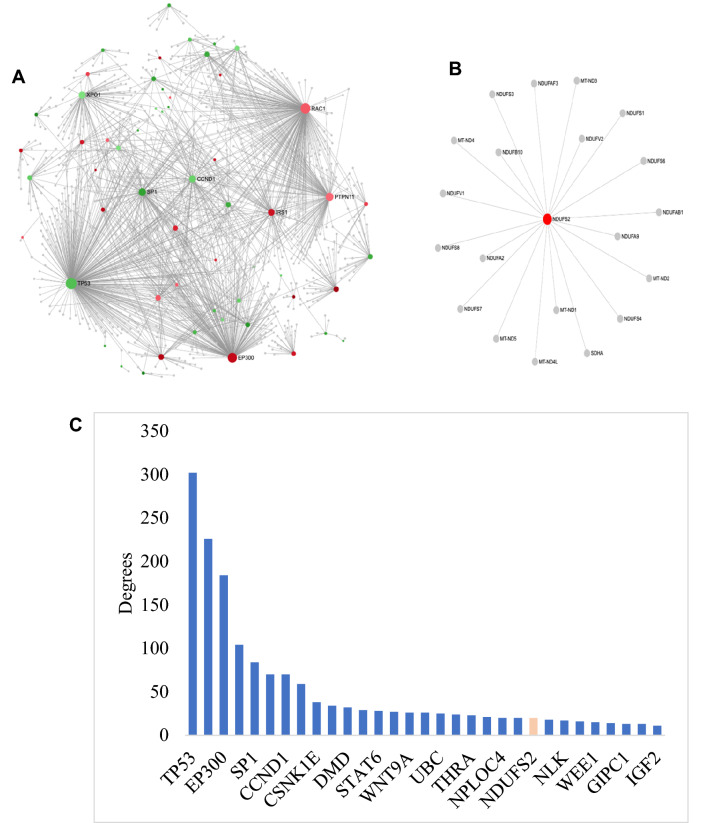
First and second networks of EJ28Pi cells. (**A**) First identified network of EJ28Pi cells. The red and green colours represent the nodes expression that are up- and down-regulated, respectively. The expression levels are represented by colour and the area of the nodes indicate the degrees in which the nodes are connected to each other. Nodes with gene names are the top 8 nodes in the PPI network. (**B**) Second identified network of EJ28Pi cells. Subnetwork 2 shows only the upregulated node that is affected in the pathway. The red colour node is is upregulated in EJ28Pi cells. (**C**) Hub nodes in the PPI network of EJ28Pi cells. The top 16 hub nodes with their degree of connection levels are shown. Genes in blue colour are from subnetwork 1 and red from subnetwork 2. The PPI network figures A & B are generated using a multifunctional online software, Network Analyst (https://www.networkanalyst.ca)^23,24^.

Subsequently, the 134 DEGs associated with EJ28Pi cells were analysed for protein–protein network interactions. A network comprising of 14 subnetworks including one continent (subnetwork 1) and 13 islands (subnetwork 2–14) was identified. The network with the highest scores were selected and analysed in order to provide an insight into the mechanisms associated with the development of NDV persistent infection in EJ28 cells. Subnetwork 1 had 1161 nodes, 1662 edges and 57 seeds (Fig. 4A), while subnetwork 2 had 21 nodes, 20 edges and 1 seed (Fig. 4B). The expression of each node is represented by different colours while the degree of connection between nodes are represented by the area. The top 16 hub nodes from the entire network analysis were assessed for their distribution and 15 hub nodes out of this total were from subnetwork 1. Only NADH ubiquinone oxidoreductase core subunit S2 (*NDUFS2)* was from subnetwork 2 (Fig. 4C).

**Figure 5:**
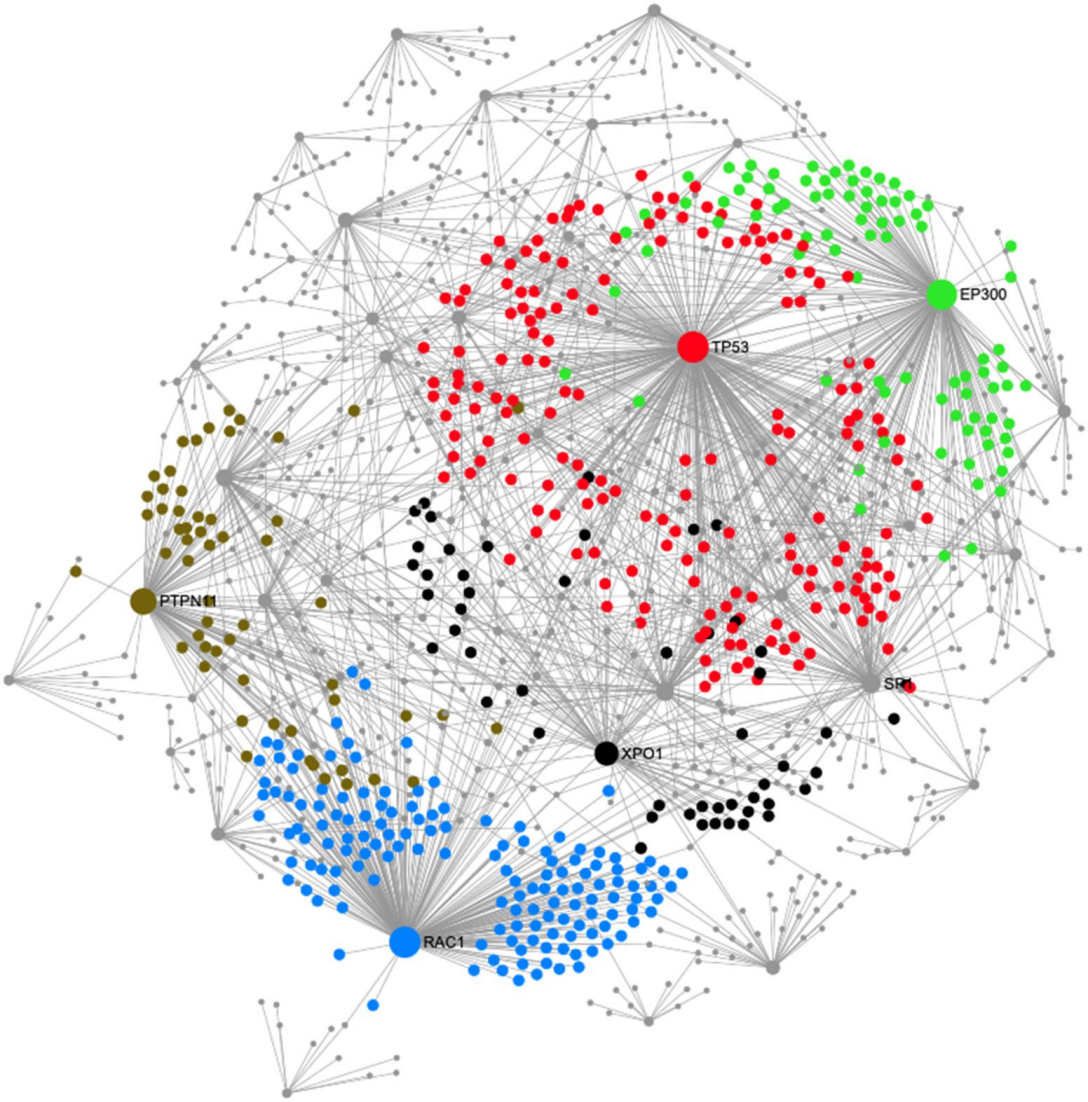
Modules 4, 5, 6, 7 and 8 of the EJ28Pi PPI network. The modules in red, blue, green, black and brown are for modules 4, 5, 6, 7, and 8, respectively. The degrees of connection between nodes in the network are represented by the areas of the nodes. The PPI network figure was generated using a multifunctional online software, Network Analyst (https://www.networkanalyst.ca)^23,24^.

Next, functional connections within the constructed network were studied and the related nodes were re-designed (Fig. 5). As shown, pathways of renal cell carcinoma, viral carcinogenesis, proteoglycans in cancer, prostate cancer, insulin resistance, Ras signalling, circadian rhythm, neurotrophin signalling, cell cycle, aldosterone-regulated sodium reabsorption, FoxO signalling, microRNAs in cancer, Wnt signalling, influenza A, tight junction, viral myocarditis, Kaposi's sarcoma-associated herpesvirus infection, PI3K-Akt signalling, epithelial cell signaling in Helicobacter pylori infection, adipocytokine signalling, epstein-Barr virus infection, adherens junction, bacterial invasion of epithelial cells, cAMP signalling, HTLV-I infection, Longevity regulating pathway, glucagon signaling pathway, leukocyte transendothelial migration, AMPK signaling pathway, pathways in cancer, natural killer cell mediated cytotoxicity and measles were significantly enriched (*p* < 0.05) in the nodes containing upregulated DEGs (subnetwork 1). The complete list of these pathways and their false discovery rates (FDRs) are listed in Supplementary Table S3.

Meanwhile, pathways of Wnt signaling, proteoglycans in cancer, HTLV-I infection, cancer, breast cancer, cellular senescence, melanoma, glioma, MAPK signaling, transcriptional misregulation in cancer, focal adhesion, prostate cancer, endocrine resistance, Th17 cell differentiation, thyroid hormone signaling, thyroid cancer, FoxO signaling, bladder cancer, measles, oxytocin signaling, hippo signaling, amyotrophic lateral sclerosis (ALS), hepatitis C, Jak-STAT signaling, hepatitis B, endometrial cancer, basal cell carcinoma, axon guidance, mitophagy—animal, central carbon metabolism in cancer, Inflammatory bowel disease (IBD), non-small cell lung cancer, Kaposi's sarcoma-associated herpesvirus infection, long-term potentiation, amphetamine addiction, PI3K-Akt signaling, p53 signaling, platinum, drug resistance, pancreatic cancer, chronic myeloid leukemia, regulation of actin cytoskeleton, colorectal cancer, Th1 and Th2 cell differentiation, small cell lung cancer, choline metabolism in cancer, neurotrophin signaling pathway, cell cycle, platelet activation and oocyte meiosis were significantly enriched in the nodes containing the downregulated DEGs (subnetwork 1). The identified pathways with their false discovery rates (FDRs) can be found in Supplementary Table S4.

The PPI network consisted of 41 modules but only modules with majority of the nodes were selected for redesigning of the modular network. Modules 4, 5, 6, 7, and 8 had the most significant number of nodes (*p* < 0.05) that contributed to the activation and enrichment of the aforementioned pathways. The modules are coloured red (module 4), blue (module 5), green (module 6), black (module 7) and brown (module 8) respectively (Fig. 5). These five modules have been identified as significantly (*p* < 0.05) acting together to contribute towards the development of NDV persistent infection in EJ28 bladder cancer cells; by virtue of their functionally connections via the upregulation of *EP300*, *IRS1*, *PTPN11*, and *RAC1*and downregulation of *TP53*, *SP1*, *CCND1* and *XPO1* respectively.

**Figure 6:**
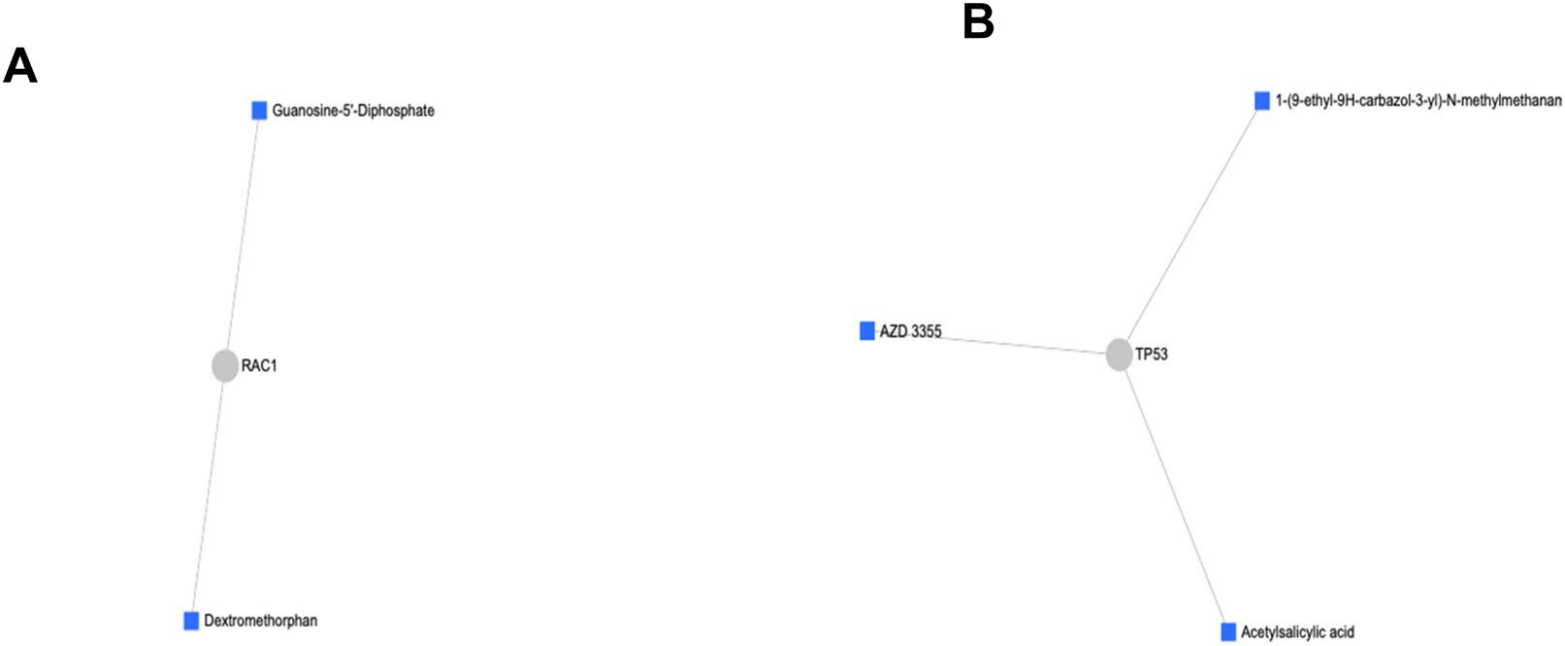
Protein-drug interaction network. (**A**) Interactions between the upregulated node (*RAC1*) and two drugs. (**B**) Interactions between upregulated node (*TP53*) and three drugs.

Protein-drug interaction network analysis was performed. The upregulated nodes that include *EP300*, *IRS1*, *PTPN11*, and *RAC1,* as well as the downregulated nodes that include*TP53*, *SP1*, *CCND1* and *XPO1* were mapped to the DrugBank database for matching nodes to obtain specific drug interaction information. Two subnetworks were discovered with subnetwork 1 containing 4 nodes, 3 edges and 1 seed and subnetwork 2 containing 3 nodes, 2 edges and 1seed. Dextromethorphan and guanosine-5'-diphosphate drugs were identified to effectively interact with the upregulated rac family small GTPase 1 (*RAC1*) node (Fig. 6A). In subnetwork 2, tumour protein P53 (*TP53*) that was downregulated is connected to acetylsalicylic acid, AZD, and 1-(9-ethyl-9H-carbazol-3-yl)-N-methylmethanamine drugs (Fig. 6B), which basically means that these drugs can be potentially used together with NDV to enhance the oncolytic activity of NDV against EJ28 bladder cancer cell lines and prevent the cells from acquiring persistent infection.

### Validation of hub genes in Oncomine

**Figure 7:**
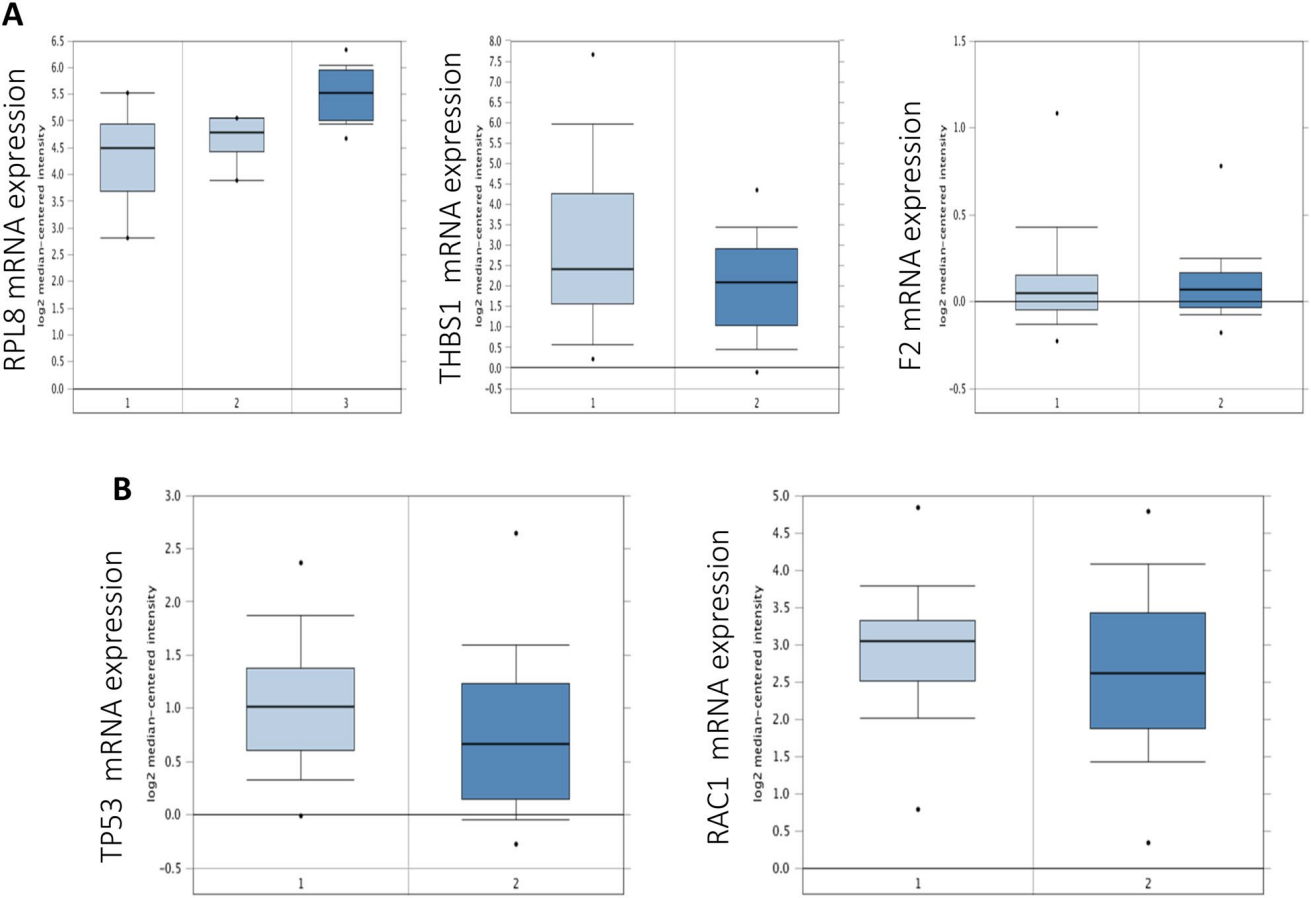
Expression of *RPL8*, *THBS1, F2, TP53,* and *RAC1* mRNA levels obtained from TCCSUPPi and EJ28Pi. (**A**) Expression of *RPL8*, *THBS1* and *F2* mRNA levels obtained from TCCSUPPi in bladder cancer using Oncomine. (**B**) Expression of *TP53* and *RAC1* mRNA levels obtained from EJ28Pi in bladder cancer using Oncomine. Left plot represents the expression in normal bladder tissue while the right plot represents the expression in bladder cancer tissue.

To validate the expression profiles of the top hub genes that were identified in the protein–protein interaction networks, mRNA expression mining of these hub genes in publicly available Oncomine database (www.oncomine.org)^25^ that included the upregulated nodes *RPL8* and *THBS1* as well as the downregulated nodes *F2* in TCCSUPPi cells was carried out. Likewise, the upregulated node *RAC1* and downregulated node *TP53* obtained from EJ28Pi cells were subsequently investigated. The results show that among the three hub genes (*RPL8, THBS1* and *F2*) obtained from TCCSUPPi cells, *RPL8* was significantly upregulated in bladder cancer cells as compared to normal bladder tissue (GSE3167; Fig. 7 upper left; *p* = 6.36E−5)^26^. In a study undertaken by Kim, Kim^27^, relative expression of *THBS1* and *F2* were slightly higher in bladder cancer than in the normal bladder tissue but the difference was not statistically significant (GSE13507; Fig. 7 upper middle & upper right; *p* > 0.05), suggesting that *RPL8, THBS1* and *F2* genes potentially play a vital role in bladder cancer progression and metastasis. These genes may also be the reason why cancer cells are able to maintain normal growth and development even after NDV infection as seen in the case of the established persistent TCCSUPPi cells.

*TP53* and *RAC1* mRNA expression levels in bladder cancer tissues were shown to be lower than that in the normal bladder tissue (GSE13507; Fig. 7 lower left & lower right; *p* > 0.05). The data suggest that *TP53* and *RAC1* are downregulated in bladder cancer and may likely contribute towards the development of NDV persistent infection in EJ28 cells; noting that *TP53* was actually found to be upregulated in the established persistent EJ28Pi cells.

## Discussion

Analysis of the RNA-Seq data revealed several essential pathways that were enriched by the DEGs associated with NDV persistent infection in bladder cancer cell lines. We then performed protein–protein interaction (PPI) analysis of the identified DEGs to unravel the connections between these pathways and the specific genes. Functional connectivity in the biological processes was observed through this analysis. By modular analysis of the PPI network of persistent TCCSUPPi cells, the *RPL8* node group was upregulated and implicated in the pathways of bladder cancer, malaria, mitophagy, p53 signaling, ECM-receptor interaction, TGF-beta signaling, phagosome, ribosome, focal adhesion and proteoglycans in cancer were clustered in the same module, suggesting that these genes co-function together to develop NDV persistent infection in the TCCSUP bladder cancer cell line. *RPL8*, codes for the ribosomal protein L8 that is known to be associated with viral mRNA translation and rRNA processing pathways in both the nucleus and the cytosol^28^. In our study, *RPL8* gene was upregulated in the persistent TCCSUPPi cells. Dysregulation or misexpression of this gene family is associated with different types of cancer cells^29–32^ and several other inherited genetic diseases^33^. This shows that *RPL8* is associated with viral infection and its role in establishing NDV persistent infection in TCCSUP bladder cancer cell line is biologically significant in this study.

On the other hand, the upregulated nodes found in persistent EJ28Pi cells include *EP300, PTPN11*, and *RAC1 *that are implicated in the pathways of renal cell carcinoma, viral carcinogenesis, proteoglycans in cancer, prostate cancer, insulin resistance, Ras signalling, circadian rhythm, neurotrophin signalling, cell cycle, aldosterone-regulated sodium reabsorption, FoxO signalling, microRNAs in cancer, Wnt signalling, influenza A, tight junction, viral myocarditis, Kaposi's sarcoma-associated herpesvirus infection, PI3K-Akt signalling, epithelial cell signalling in helicobacter pylori infection, adipocytokine signalling, epstein-Barr virus infection, adherens junction, bacterial invasion of epithelial cells, cAMP signalling, HTLV-I infection, longevity regulating pathway, glucagon signalling pathway, leukocyte transendothelial migration, AMPK signalling pathway, pathways in cancer, natural killer cell mediated cytotoxicity and measles, were clustered in module 5, 6, and 7 (Fig. 4), indicating that the genes in these cluster act together to facilitate the development of NDV persistent infection in EJ28 bladder cancer cell line. Our findings demonstrate that *EP300*, *PTPN11*, and *RAC1* genes were upregulated in persistent EJ28Pi cells. *EP300* gene is known to encode for adenovirus E1A-associated cellular p300 transcriptional co-activator protein that functions in the regulation of transcription by remodeling chromatin^34–36^. It plays a vital role in cell proliferation, differentiation and epithelial cancer^37^ as well as co-activates hypoxia-inducible factor 1 alpha, *HIF1A* to facilitate the expression of VEGF, a key player in inducing cellular hypoxia^38^. A recent study suggested that HAT domain mutation on *EP300* gene has a major impact on malignant progression and growth^39^, suggesting that *EP300* gene may contribute to the survival and growth of EJ28 cells after they are persistently infected with oncolytic NDV.

*PTPN11* also known as protein tyrosine phosphatase, none-receptor type 11 is an oncogene that has the potential to turn normal cells into cancerous cells when mutated. It encodes a protein that is in the protein tyrosine phosphatase (PTP) family and provides key important functions in cell growth and differentiation. *PTPN11* gene is mutated in about 1.19% of all types of cancers including melanoma, carcinoma of the lung, malignant glioma and leukaemia^40^. This is consistent with our findings that demonstrate that *PTPN11* gene is upregulated in NDV persistent EJ28Pi cells. In addition, *RAC1* RAS superfamily was also found to be upregulated in EJ28Pi cells. It functions by controlling cytoskeletal reorganization and cell growth. In line with our results, previous evidence has shown that the upregulation of *RAC1* is associated with lymphovascular invasion and lymph node metastasis of the urinary tract cancer^41^. Therefore, the upregulation of *EP300, PTPN11*, and *RAC1* genes may play a role in the development of NDV persistent infection in EJ28 bladder cancer cell line.

Protein-drug interaction network analyses revealed drugs that may be used to disrupt the linkages between the major modules in the constructed PPI networks of both the TCCSUPPi and EJ28Pi cells. We observed several drugs that are linked to the downregulated node *F2* in TCCSUPPi cells that includes lepirudin, bivalirudin, drotrecogin alfa, coagulation factor IX (recombinant), menadione, argatroban and proflavine etc. On the other hand, puromycin, anisomycin and alpha-hydroxyl-beta-phenyl-propionic acid were the drug targets for the upregulated *RPL8* node group. One important drug among the connected drugs on *F2* node network is menadione, a biologically active vitamin K3 that promotes coagulation of blood and is reported to strongly inhibit malignant growth, especially in neoplastic cells via the conversion to vitamin K2 as well as reducing in mutagenesis risk in rapidly growing cells of newborns^42^. Other studies have demonstrated synergistic effects of vitamin C and menadione (vitamin K3) alone with radiotherapy in killing bladder cancer cells as well as other cancer types without adverse reactions to patients^43–45^. Anisomycin is an antibiotic that inhibits the synthesis of proteins via the activity of peptidyl transferase in ribosomes^46^. It interacts with the *RPL8* node in the TCCSUPPi PPI network. Studies have found that this drug kills a variety of cancer cells such as colorectal and leukaemia through the induction of apoptosis^47,48^. Thus, anisomycin that interacts with *RPL8* as well as menadione and the remaining drugs that interacts with *F2* are drugs that can potentially reverse the mechanisms employed by TCCSUP cells in acquiring NDV persistent infection. They may potentially be used in combination with NDV to synergistically kill bladder cancer cells and prevent the acquiring of persistency of infection.

The identified drugs that interact with the upregulated rac family small GTPase 1 (*RAC1*) node in the EJ28Pi cells network include dextromethorphan and guanosine-5'-diphosphate. On the other hand, acetylsalicylic acid, AZD, and 1-(9-ethyl-9H-carbazol-3-yl)-N-methylmethanamine were found to interact with the downregulated *TP53* node. Dextromethorphan belongs to a class of drugs known as morphinan that has sedative and stimulant properties. It is often used as a cough suppressant. Dextromethorphan is found to be capable of causing mild motor and cognitive impairment, paranoia and delusion^49^. It is unclear how dextromethorphan exerts its effects on bladder cancer cell lines. Thus, investigating the role of dextromethorphan in bladder cancer cell lines is needed to understand its mechanism of action in cancer. We also found that guanosine-5'-diphosphate drug interacts with the *RAC1* network. It has been reported that guanosine-5'-diphosphate in combination with other drugs can significantly reduce the aggressive nature of breast cancers^50^. Another important drug identified to interact with the *TP53* node in the network is AZD. This drug may potentially be used for bladder cancer treatment because AZD is already being used in clinical trials, testing against many different types of cancers including esophageal cancer^51^, small cell lung cancer^52^ and colorectal cancer^53^. Therefore, the drugs mentioned above may be used in combination with NDV to kill EJ28 bladder cancer cells and prevent them from acquiring NDV persistent infection.

Our analyses also discovered that some upstream genes such as *RPL8* and *F2* were significantly upregulated in bladder cancer with the exception of *THBS1, TP53* and *RAC1* that were found to be less expressed according to the Oncomine-based expression analysis. Through PPI network analysis, we identified *RPL8* and *F2* as therapeutic drug targets that were significantly overexpressed in bladder cancer. The expression patterns indicate that *RPL8*, *F2, THBS1, TP53* and *RAC1* are differentially expressed in bladder cancer as compared to normal bladder tissues and that the level of their expression varies depending on stage of tumourigenesis.

## Conclusion

This study presents the novel analysis of the transcriptomic profiles of NDV persistent bladder cancer cell lines to construct protein–protein interaction (PPI) networks. We observed that the upregulated *RPL8* group and downregulated *HSPA1A/HSPA4* group are functionally connected with one another based on the data from TCCSUPPi cells. Through the *RPL8*-*HSPA1A/HSPA4* link, a considerable number of pathways were identified that include bladder cancer, malaria, p53 signaling, ECM-receptor interaction and TGF-beta signaling pathways that in turn, indicate to the molecular pathways associated with NDV persistent infection in TCCSUP bladder cancer cells. Similarly, we identified the functional connectivity between upregulated *EP300, IRS1*, *PTPN11*, and *RAC1* and the downregulated *TP53*, *SP1*, *CCND1* and *XPO1*  in EJ28Pi cells. Through *IRS1*, *PTPN11*, and *RAC1* as well as *TP53*, *SP1*, *CCND1* and *XPO1* connections, several major pathways including renal cell carcinoma, viral carcinogenesis, cell cycle, FoxO signaling, pathway in cancer, NK cells mediated cytotoxicity, Ras signaling pathways were implicated as key molecular pathways associated with NDV persistent infection in EJ28 cells.

Furthermore, our data analyses provide clues into identifying new drug targets that can synergistically act together with NDV to effectively kills bladder cancer cells as well as reverse or prevent the ability of these cells to acquire NDV persistent infection. The results in this study therefore enhances our understanding of host responses to NDV persistent infection by identifying putative candidate genes and pathways that are associated with persistent infection in bladder cancer cells. In addition, several proteins that are crucial to the development of persistent infection were also identified through the PPI networks.

Finally, we recommend that the drugs identified to target the key nodes on the PPI network should be screened functionally as this will help to identify new approaches for cancer treatment in combination with the oncolytic Newcastle disease virus. Overall, this work provides the molecular basis for further research to unravel the function of these novel pathways, candidate genes and specific proteins that seems to activate the phenotypes associated with NDV persistent infection in bladder cancer cells.

## Materials and methods

### DEGs from persistently infected cells

The DEGs in this study were previously published in a preprint^13^ and deposited in NCBI Gene Expression Omnibus (GEO) under accession number GSE140902 and released to the public (https://www.ncbi.nlm.nih.gov/geo/query/acc.cgi?acc=GSE140902). A total of 63 and 134 differentially expressed genes (DEGs) from persistent TCCSUPPi and EJ28Pi cells relative to their uninfected controls were obtained from the total RNA samples of these NDV persistently infected cells. Of the 63 DEGs in TCCSUPPi cells, 25 genes were upregulated and 38 genes were downregulated. Whilst in EJ28Pi, 55 genes were upregulated and 79 genes were downregulated. These DEGs were enriched into pathways as reported in the paper and were used for the PPI network construct and analysis.

### PPI network analysis

DEGs obtained from the persistently infected cells were used to construct the PPI network using a multifunctional online software, Network Analyst (https://www.networkanalyst.ca)^23,24^. Path and module analyses as well as the determination of the protein-drug interactions were carried out using this software. PPI networks were generated for each of the persistently infected cell lines by submitting the number of DEGs together with their Ensembl gene IDs and Log_2_ (fold change) expression to the NetworkAnalyst software. Search Tool for the Retrieval of Interacting Genes/Proteins (STRING) Interactome was used as the PPI database and the cut off confidence scores was set at 900 since the range of scores were between 400 to 1000^22^. The seeds were mapped to the corresponding molecular interaction database and the subnetworks with the most cluster of nodes were chosen and identified as top nodes in the network.

### Nodal path exploration

Nodes that are functionally connected and linked in the generated PPI network were visualized using the path explorer function segment of the NetworkAnalyst software^23^. Paths of interest and the connections between nodes were selected and redesigned to enable further understanding of the cascades of events in the PPI network. The implicated nodes that were significantly enriched in the pathways that were associated with the development of persistent infection were specifically marked in the connections.

### Exploring module in the PPI network

Using the same NetworkAnalyst software^23^, a module explorer section of the application was used to identify the cluster of subnetworks that collectively function together in the PPI networks through Walktrap Algorithm that uses a walk-based strategy to detect modules. The degrees of internal (edges within a module) and external (edges connecting the nodes of a module with the rest of the graph) edges for determining the significance of the module was based on the Wilcoxon rank-sum and values of *p* < 0.05 were considered significant. Then, the modules of interest were marked with different colours in the PPI network.

### Protein drug interaction

The upregulated and downregulated nodes of interest obtained from persistently infected TCCSUPPi (upregulated *RPL8* and downregulated *HSPA1A*/*HSPA4* groups) and EJ28Pi (upregulated *EP300*, *IRS1*, *PTPN11*, and *RAC1* and downregulated *TP53*, *SP1*, *CCND1* and *XPO1*) cells were further analyzed to identify their interactions with drugs. DrugBank database version 5.0 was used to match the these nodes against a panel of drug targets to generate and collect the protein-drug interactions network information^54^.

### Validation of hub genes

The candidate hub genes identified from the hub module in the network were validated by an Oncomine database (http://www.oncomine.org/)^25^ The expression levels of these hub genes in both persistently infected cell lines were validated using Dyrskjøt, Kruhøffer^26^ (GSE3167) and Kim, Kim^27^ (GSE13507) gene expression data with defined thresholds values for fold change > 2, gene rank top 10% and *p* < 0.001. Data type was restricted to only mRNA expression and statistical significance was based on Student’s *t* test.

## Supplementary Information

Supplementary Information.

## Data Availability

The authors declare that all the data in this manuscript are available. Raw and processed data were deposited in NCBI Gene Expression Omnibus (GEO) under accession number GSE140902 (https://www.ncbi.nlm.nih.gov/geo/query/acc.cgi?acc=GSE140902).
