# Supplementary material for: Analysis of PPI networks of transcriptomic expression identifies hub genes associated with Newcastle disease virus persistent infection in bladder cancer
Source: Sci Rep. 2023 May 5;13:7323. doi: 10.1038/s41598-022-20521-z (PMC10162992; doi:10.1038/s41598-022-20521-z)
Supplement: Supplementary file 1 — Supplementary Information. [file 41598_2022_20521_MOESM1_ESM.docx]

**Supplementary information**

**Supplementary Table 1: List of significantly enriched pathways based on upregulated DEGs connecting the nodes in subnetwork 1 for TCCSUPPi.**

| **S/No.** | **Pathway** | **Total** | **Hits** | **P.Value** | **FDR** |
| --- | --- | --- | --- | --- | --- |
| 1 | Bladder cancer | 41 | 1 | 0.0158 | 1 |
| 2 | Malaria | 49 | 1 | 0.0189 | 1 |
| 3 | Mitophagy - animal | 65 | 1 | 0.025 | 1 |
| 4 | p53 signaling pathway | 72 | 1 | 0.0277 | 1 |
| 5 | ECM-receptor interaction | 82 | 1 | 0.0315 | 1 |
| 6 | TGF-beta signaling pathway | 92 | 1 | 0.0353 | 1 |
| 7 | Phagosome | 152 | 1 | 0.0578 | 1 |
| 8 | Ribosome | 153 | 1 | 0.0582 | 1 |
| 9 | Focal adhesion | 199 | 1 | 0.0752 | 1 |
| 10 | Proteoglycans in cancer | 201 | 1 | 0.0759 | 1 |

**Supplementary Table 2: List of significantly enriched pathways based on downregulated DEGs connecting the nodes in subnetwork 1 for TCCSUPPi.**

| **S/N.** | **Pathway** | **Total** | **Hits** | **P.Value** | **FDR** |
| --- | --- | --- | --- | --- | --- |
| 1 | Antigen processing and presentation | 77 | 3 | 3.76E-06 | 0.0012 |
| 2 | Protein processing in endoplasmic reticulum | 165 | 2 | 0.00264 | 0.419 |
| 3 | Prion diseases | 35 | 1 | 0.018 | 1 |
| 4 | Legionellosis | 55 | 1 | 0.0281 | 1 |
| 5 | Longevity regulating pathway - multiple species | 62 | 1 | 0.0317 | 1 |
| 6 | Complement and coagulation cascades | 79 | 1 | 0.0402 | 1 |
| 7 | Chagas disease (American trypanosomiasis) | 103 | 1 | 0.0522 | 1 |
| 8 | Toxoplasmosis | 113 | 1 | 0.0572 | 1 |
| 9 | Platelet activation | 124 | 1 | 0.0626 | 1 |
| 10 | Spliceosome | 134 | 1 | 0.0675 | 1 |

**Supplementary Table 3 : List of significantly enriched pathways based on upregulated DEGs connecting the nodes in subnetwork 1 for EJ28Pi.**

| **S/No.** | **Pathway** | **Total** | **Hits** | **P.Value** | **FDR** |
| --- | --- | --- | --- | --- | --- |
| 1 | Renal cell carcinoma | 69 | 4 | 2.52E-05 | 0.00802 |
| 2 | Viral carcinogenesis | 201 | 5 | 0.000127 | 0.0202 |
| 3 | Proteoglycans in cancer | 201 | 4 | 0.00154 | 0.127 |
| 4 | Prostate cancer | 97 | 3 | 0.00187 | 0.127 |
| 5 | Insulin resistance | 108 | 3 | 0.00254 | 0.127 |
| 6 | Ras signaling pathway | 232 | 4 | 0.00261 | 0.127 |
| 7 | Circadian rhythm | 31 | 2 | 0.00282 | 0.127 |
| 8 | Neurotrophin signaling pathway | 119 | 3 | 0.00334 | 0.127 |
| 9 | Cell cycle | 124 | 3 | 0.00375 | 0.127 |
| 10 | Aldosterone-regulated sodium reabsorption | 37 | 2 | 0.00401 | 0.127 |
| 11 | FoxO signaling pathway | 132 | 3 | 0.00447 | 0.129 |
| 12 | MicroRNAs in cancer | 299 | 4 | 0.00649 | 0.172 |
| 13 | Wnt signaling pathway | 158 | 3 | 0.00738 | 0.181 |
| 14 | Influenza A | 167 | 3 | 0.0086 | 0.191 |
| 15 | Tight junction | 170 | 3 | 0.00902 | 0.191 |
| 16 | Viral myocarditis | 59 | 2 | 0.00995 | 0.198 |
| 17 | Kaposi's sarcoma-associated herpesvirus infection | 186 | 3 | 0.0115 | 0.206 |
| 18 | PI3K-Akt signaling pathway | 354 | 4 | 0.0117 | 0.206 |
| 19 | Epithelial cell signaling in Helicobacter pylori infection | 68 | 2 | 0.0131 | 0.21 |
| 20 | Adipocytokine signaling pathway | 69 | 2 | 0.0134 | 0.21 |
| 21 | Epstein-Barr virus infection | 201 | 3 | 0.0142 | 0.21 |
| 22 | Adherens junction | 72 | 2 | 0.0146 | 0.21 |
| 23 | Bacterial invasion of epithelial cells | 74 | 2 | 0.0153 | 0.212 |
| 24 | cAMP signaling pathway | 212 | 3 | 0.0164 | 0.217 |
| 25 | HTLV-I infection | 219 | 3 | 0.0179 | 0.227 |
| 26 | Longevity regulating pathway | 89 | 2 | 0.0217 | 0.266 |
| 27 | Glucagon signaling pathway | 103 | 2 | 0.0285 | 0.336 |
| 28 | Leukocyte transendothelial migration | 112 | 2 | 0.0333 | 0.378 |
| 29 | AMPK signaling pathway | 120 | 2 | 0.0378 | 0.414 |
| 30 | Pathways in cancer | 530 | 4 | 0.0439 | 0.455 |
| 31 | Natural killer cell mediated cytotoxicity | 131 | 2 | 0.0443 | 0.455 |
| 32 | Measles | 138 | 2 | 0.0487 | 0.484 |

**Supplementary Table 4 : List of significantly enriched pathways based on downregulated DEGs connecting the nodes in subnetwork 1 for EJ28Pi.**

| **S/No.** | **Pathway** | **Total** | **Hits** | **P.Value** | **FDR** |
| --- | --- | --- | --- | --- | --- |
| 1 | Wnt signaling pathway | 158 | 5 | 6.63E-05 | 0.0211 |
| 2 | Proteoglycans in cancer | 201 | 5 | 0.000207 | 0.0327 |
| 3 | HTLV-I infection | 219 | 5 | 0.000308 | 0.0327 |
| 4 | Pathways in cancer | 530 | 7 | 0.000469 | 0.0373 |
| 5 | Breast cancer | 147 | 4 | 0.000701 | 0.0425 |
| 6 | Cellular senescence | 160 | 4 | 0.000963 | 0.0425 |
| 7 | Melanoma | 72 | 3 | 0.00105 | 0.0425 |
| 8 | Glioma | 75 | 3 | 0.00118 | 0.0425 |
| 9 | MAPK signaling pathway | 295 | 5 | 0.0012 | 0.0425 |
| 10 | Transcriptional misregulation in cancer | 186 | 4 | 0.00168 | 0.0536 |
| 11 | Focal adhesion | 199 | 4 | 0.00216 | 0.0623 |
| 12 | Prostate cancer | 97 | 3 | 0.00247 | 0.0623 |
| 13 | Endocrine resistance | 98 | 3 | 0.00255 | 0.0623 |
| 14 | Th17 cell differentiation | 107 | 3 | 0.00327 | 0.0743 |
| 15 | Thyroid hormone signaling pathway | 116 | 3 | 0.00411 | 0.0871 |
| 16 | Thyroid cancer | 37 | 2 | 0.00484 | 0.0962 |
| 17 | FoxO signaling pathway | 132 | 3 | 0.0059 | 0.105 |
| 18 | Bladder cancer | 41 | 2 | 0.00592 | 0.105 |
| 19 | Measles | 138 | 3 | 0.00667 | 0.112 |
| 20 | Oxytocin signaling pathway | 153 | 3 | 0.00886 | 0.127 |
| 21 | Hippo signaling pathway | 154 | 3 | 0.00902 | 0.127 |
| 22 | Amyotrophic lateral sclerosis (ALS) | 51 | 2 | 0.00905 | 0.127 |
| 23 | Hepatitis C | 155 | 3 | 0.00918 | 0.127 |
| 24 | Jak-STAT signaling pathway | 162 | 3 | 0.0104 | 0.134 |
| 25 | Hepatitis B | 163 | 3 | 0.0105 | 0.134 |
| 26 | Endometrial cancer | 58 | 2 | 0.0116 | 0.142 |
| 27 | Basal cell carcinoma | 63 | 2 | 0.0136 | 0.143 |
| 28 | Axon guidance | 181 | 3 | 0.014 | 0.143 |
| 29 | Mitophagy - animal | 65 | 2 | 0.0144 | 0.143 |
| 30 | Central carbon metabolism in cancer | 65 | 2 | 0.0144 | 0.143 |
| 31 | Inflammatory bowel disease (IBD) | 65 | 2 | 0.0144 | 0.143 |
| 32 | Non-small cell lung cancer | 66 | 2 | 0.0148 | 0.143 |
| 33 | Kaposi's sarcoma-associated herpesvirus infection | 186 | 3 | 0.015 | 0.143 |
| 34 | Long-term potentiation | 67 | 2 | 0.0153 | 0.143 |
| 35 | Amphetamine addiction | 68 | 2 | 0.0157 | 0.143 |
| 36 | PI3K-Akt signaling pathway | 354 | 4 | 0.0164 | 0.145 |
| 37 | p53 signaling pathway | 72 | 2 | 0.0175 | 0.15 |
| 38 | Platinum drug resistance | 73 | 2 | 0.018 | 0.15 |
| 39 | Pancreatic cancer | 75 | 2 | 0.0189 | 0.154 |
| 40 | Chronic myeloid leukemia | 76 | 2 | 0.0194 | 0.154 |
| 41 | Regulation of actin cytoskeleton | 214 | 3 | 0.0218 | 0.169 |
| 42 | Colorectal cancer | 86 | 2 | 0.0244 | 0.185 |
| 43 | Th1 and Th2 cell differentiation | 92 | 2 | 0.0277 | 0.204 |
| 44 | Small cell lung cancer | 93 | 2 | 0.0282 | 0.204 |
| 45 | Choline metabolism in cancer | 99 | 2 | 0.0317 | 0.224 |
| 46 | Neurotrophin signaling pathway | 119 | 2 | 0.0444 | 0.307 |
| 47 | Cell cycle | 124 | 2 | 0.0478 | 0.315 |
| 48 | Platelet activation | 124 | 2 | 0.0478 | 0.315 |
| 49 | Oocyte meiosis | 125 | 2 | 0.0485 | 0.315 |
